# The experıance of tertıary center for adult granulosa cell tumor: whıch factors predıct survival?

**DOI:** 10.1186/s13048-024-01453-w

**Published:** 2024-06-19

**Authors:** Mustafa Şahin, Tufan Arslanca, Yeşim Özkaya Uçar, Gülşah Tiryaki Güner, İlker Selçuk, Hakan Raşit Yalçın

**Affiliations:** Department of Gynecological Oncology Surgery, Ankara Bilkent City Hospital, Universiteler Mahallesi, 1604. Cadde No: 9. Çankaya, Ankara, Türkiye

**Keywords:** Adult granulosa cell tumors, Prognostic factors, Cytoreductive surgery, Recurrent tumors

## Abstract

**Background:**

This retrospective study aims to evaluate the clinical course and long-term outcomes of patients diagnosed with adult granulosa cell tumors (AGCT).

**Methods:**

The study analyzed a cohort of 112 AGCT patients with a median follow-up of 87 months. Data regarding disease-free survival (DFS), overall survival (OS), recurrence rates, and prognostic factors were collected and analyzed. Surgical interventions, including lymphadenectomy and cytoreductive surgery, were assessed for their impact on outcomes.

**Results:**

The study revealed favorable long-term outcomes, with a 5-year DFS of 85% and a 10-year DFS of 83%. Additionally, a 5-year OS of 100% and a 10-year OS of 96% were observed. Recurrence occurred in 13.4% of cases, with advanced stage and positive peritoneal cytology identified as independent poor prognostic factors for DFS. Lymph node involvement was rare, and routine lymphadenectomy did not improve outcomes. Conservative surgery showed comparable DFS rates to definitive surgery in early-stage disease. However, cytoreductive surgery was crucial for advanced and recurrent tumors, with complete tumor resection enhancing survival outcomes.

**Conclusion:**

The study underscores the importance of vigilant follow-up and individualized treatment strategies for AGCT patients. Despite the retrospective nature of the analysis, the substantial patient cohort and meticulous surgical interventions contribute valuable insights into AGCT management. Prospective multicenter studies are warranted to further elucidate prognostic factors and optimize treatment approaches for this rare malignancy.

## Background

Granulosa cell tumors are the most common malignant sex cord stromal tumors (SCST), accounting for approximately 2–5% of all ovarian malignancies with an incidence rate of approximately 0.4–1.7/100.000 [1]. Granulosa cell tumors are divided into two histological types; adult granulosa cell tumors (AGCT) and juvenile granulosa cell tumors. Approximately 95% of all patiens possess the adult variant. Although, AGCT is usually diagnosed in the premenopausal or early postmenopausal period, it can be seen at younger ages as well. AGCT has a favorable prognosis and shows a slow clinical course. The 5-year overall survival rate is 75–95% in stage I, 55–75% in stage II, and 22–50% in stage III/IV. The tumor stage is the most significant factor associated with oncologic outcome [1, 2]. Recurrences in AGCT are mostly multifocal and the most common site of recurrence is the pelvis. Recurrence rates range from 6 to 48% and 50–80% of patients who have a mortal course. Recurrences are most common within 5 years following surgery, while late recurrences can be observed after 30–40 years [3, 4]. Due to the slow growth of the tumor and hormonal symptoms, most patients are diagnosed in the early stages [5].

The primary treatment for AGCT is surgery, which can be curative in the early stages. In the postmenopausal period, the standard treatment is a hysterectomy and a bilateral salpingo-oophorectomy. In early-stage patients who wish to preserve their fertility, conservative surgery that preserves the other ovary and uterus can be performed [6]. The role of surgical staging in the treatment of AGCT is still unclear. The benefit of lymphadenectomy is controversial and only the removal of suspicious lymph nodes is recommended [7].

The National Comprehensive Cancer Network (NCCN) guideline recommends adjuvant chemotherapy in advanced stages. However, there is no evident consensus on adjuvant treatment in stage 1C [8]. The role of adjuvant chemotherapy in the treatment of primary or recurrent disease in AGCT is still unclear [2].

The rarity of AGCT makes it difficult to recognize the prognostic factors, predict the oncologic outcomes and determine the appropriate treatment. In this study, we aimed to investigate the clinicopathologic prognostic factors affecting the recurrence and survival in AGCT patients.

## Materials and methods

The data of 112 patients who have been diagnosed and treated for AGCT between the years of January 2004 and August 2019 in the gynecologic oncology clinic were retrospectively evaluated. Data was obtained from the electronic database system, patient files, pathology reports and operative notes. Ethics committee approval has been obtained for the study (decision number 14 dated April 27, 2021).

The International Federation of Gynecology and Obstetrics (FIGO) 2014 staging system was used for staging [9]. Those operated on before 2014 were re-staged according to 2014 FIGO criteria, by re-evaluating the pathology reports. The extent of the first operation was evaluated according to the extent of the disease and the desire for fertility. The surgical procedure, in which at least part of the one ovary and uterus were preserved, was defined as “conservative surgery”. In our center, conservative treatment is applied to patients with stage IA, 1B and stage IC1 according to the 2014 FIGO staging system, and to patients with fertility potential and close follow-up opportunity. Patients with fertility desires underwent unilateral salpingo-oophorectomy without hysterectomy. “Definitive surgery” was defined as hysterectomy and bilateral salpingo-oophorectomy. The inclusion of lymphadenectomy and omentectomy in the surgical procedure was determined by the senior surgeon. The upper limit of para-aortic lymphadenectomy was the left renal vein. When evidence of a more extensive disease existed, cytoreductive surgical techniques were used, as well as, staging surgery.

The adjuvant treatment decision was made by the gynecologic oncology council. In our hospital, bleomycin, etoposide and cisplatin (BEP; 3 or 4 cycles) are most commonly preferred in the adjuvant treatment of AGCT, and platinum-based chemotherapy regimens such as carboplatin/ paclitaxel (CP; 6 cycles) or etoposide/cisplatin (EP; 6 cycles) are also used. Chemotherapy response in patients was evaluated according to RECIST 1.1 criteria [10]. Clinical responses were defined as follows: (a) Complete clinical response (CCR): Complete disappearance of lesions and absence of new lesions; (b) Partial clinical response (PCR): A reduction in the size of lesions by at least 30%; (c) Progressive disease (PD): A ≥ 20% increase in the maximum diameter of the lesion, the appearance of a new lesion ≥ 1 cm, or progression of a non-target lesion; (d) Stable disease (SD): Lesions that were neither in the partial clinical response group, nor in the progressive disease group, based on the smallest overall diameters at the time of the study. The clinical response of patients was evaluated 1 month after the first treatment (surgery + adjuvant treatment) using clinical, laboratory parameters and imaging methods.

After treatment, patients were followed up every 3 months for the first 2 years, every 6 months until the fifth year and annually thereafter. We defined recurrences distal to the pelvic inlet as pelvic recurrence, recurrences between the pelvic inlet and diaphragm as upper-abdominal recurrence, and other recurrences as extra-abdominal recurrence. Cytologically defined ascites and peritonitis carcinomatosa were considered as upper-abdominal recurrence, and recurrence in the liver parenchyma was evaluated as extra-abdominal recurrence.

## Statistical analysis

Statistical analysis was performed using SPSS version 22 (IBM, Chicago, USA). Disease-free survival (DFS) was defined as the time from operation until recurrence/progression of disease or last contact in those who did not develop recurrence. Overal survival (OS), was defined as the time from disease to death or last contact. The Kaplan–Meier method was used for survival analysis and differences were analyzed by the log-rank test. Factors with a *p* value less than 0.05 in the univariate analysis were included in the multivariate analysis. The Cox regression model was used in the multivariate analysis. The cut-off point for statistical significance was set as *p* value less than 0.05.

## Results

### Clinical, surgical, and pathological features

The mean age of the 112 patients that constituted the study group was 50.3 ± 12.57 years and ranged between 17 and 81 years of age. The mean age of the 13 patients (11.6%) that underwent conservative surgery was 30.6 ± 7.6 years and ranged between 17 and 43 years of age.

Abdominal or pelvic pain (24.1%) and palpable adnexal mass (21.4%) were the most frequently reported presenting symptoms, followed by abdominal distention (18.8%) and vaginal bleeding (17.9%). Other less common symptoms accounted for 7.1% of cases. Only 12 patients (10.7%) were asymptomatic and were diagnosed incidentally during investigations conducted for non-gynecologic reasons. The mean tumor size was 89.6 ± 55.76 mm and ranged between 10 and 300 mm. Lymphadenectomy was performed in 94 of the patients (83.9%). Pelvic and para-aortic pelvic lymph node dissection was performed in 91 patients (81.3%), and only pelvic lymph node dissection was performed in 3 patients (2.6%). The mean number of lymph nodes removed in these patients was 51 ± 26.27 and ranged between 7 and 132. Three (3.2%) of the patients who underwent lymphadenectomy had positive lymph node metastasis and the metastases were in the pelvic lymph nodes. One hundred-four (92.8%) patients were stage I, 3 (2.7%) were stage II, 4 (3.6%) were stage III, and 1 (0.9%) was stage IV. Preoperative cyst rupture was detected in 2 (1.8%) patients, and 17 (15.2%) patients had intraoperative cyst rupture. Peritoneal cytology revealed malignancy in 12 (10.7%) of the patients and 4 (4%) had metastases in the omentum. The average time from the diagnosis of the disease to the first surgery of 112 patients was 15.2 ± 10.3 days and ranged from 1 to 54 days. Complementary surgery was performed in 35 (31.2%) patients after the first surgery. The average time from diagnosis of the disease to completion surgery is 45.7 ± 11.5 days and varies between 23 and 67 days. No residue tumor was observed in all patients during initial surgery (Table 1.).

**Table 1:** Clinical, surgical, and pathological features in patients with AGCT

**Factors**		**Mean±SD**	**Median (min-max)**
Age		50.3±12.57	51 (17-81)
Tumor size (mm)		89.6±55.76	70 (10-300)
Number of harvested lymph node (total)		51±26.27	45 (7-132)
Preoperative CA 125 (IU/ml)		20.7±30.45	11 (1-174)
		***n***	**%**
FIGO stage	IA	72	64.3
	IB	9	8
	IC	23	20.5
	IIA	2	1.8
	IIB	1	0.9
	IIIA	-	-
	IIIB	2	1.8
	IIIC	2	1.8
	IV	1	0.9
Menopausal status	Premenopause	52	46.4
	Postmenopause	60	53.6
Rupture of cyst	Unruptured	90	80.4
	Iatrogenic rupture	17	15.2
	Presurgical rupture	2	1.8
	Not reported	3	2.7
Hysterectomy	Performed	93	83
	Not performed^a^	13	11.6
	Hysterectomy performed before disease	6	5.4
Lymphadenectomy	Performed	94	83.9
	Not performed	17	15.2
	Not reported	1	0.9
Lymph node metastasis^b^	Negative	89	94.7
	Positive	3	3.2
	Unknown	2	2.1
Peritoneal cytology	Negative	86	76.8
	Positive	12	10.7
	Not reported	14	12.5
Omentectomy	Performed	99	88.4
	Not performed	10	8.9
	Not reported	3	2.7
Omental metastasis^c^	Negative	95	96
	Positive	4	4
Ascites	Absent	78	69.6
	Present	15	13.4
	Not reported	19	17

^a^Conservative surgery

^b^*n*=94 patients who underwent lymphadenectomy

^c^*n*=99 patients who underwent omentectomy

### Adjuvant treatment and survival analysis

Adjuvant chemotherapy was administered to 30 patients. Of these, 22 were stage IC and 8 were stage 2–4. Of these, 17 patients (64.9%) received BEP, and 13 (35.3%) received others as adjuvant therapy (12 patients CP and 1 patient EP). While the 5-year DFS was 68% in the group receiving BEP, it was 59% in the other group (*p* = 0.773). It was observed that adjuvant treatment types did not determine DFS. Thirty patients received adjuvant therapy, with a 5-year disease-free survival (DFS) rate of 64%. Conversely, 82 patients did not receive adjuvant therapy, and their 5-year DFS rate was 94%. The 5-year DFS rate significantly decreased in patients receiving adjuvant therapy compared to those who did not (64% vs. 94%; *p* < 0.001) (Table 2).

**Table 2:** Factors related to disease-free survival in patients with AGCT

**Univariate Analysis**				**Multivariate Analysis**		
**Parameter**		**5-year Disease-Free Survival**		**Recurrence**		
		**%**	***p* Value**	**Odds Ratio**	**95% CI**	***p* Value**
Age at initial diagnosis^a^	≤51 years	85	*0.641*			
	>51 years	85				
Peritoneal cytology	Negative	93	***0.001***	Reference	1.125-16.072	***0.033***
	Positive	56		4.251		
Stage	I	91	***<0.001***	Reference	19.415-669.883	***<0.001***
	II-IV	13		114.042		
Menopausal status	Premenopause	86	*0.439*			
	Postmenopause	84				
Rupture of cyst	Unruptured	87	*0.855*			
	Ruptured^b^	82				
Ovarian tumor size^a^	≤70 mm	92	*0.055*			
	>70 mm	82				
Ascites	Absent	89	*0.689*			
	Present	87				
Surgery type	Definitive surgery	85	*0.462*			
	Conservative surgery	89				
Lymphadenectomy	Performed	87	*0.167*			
	Not performed	62				
Adjuvant treatment	Not received	94	***<0.001***			
	Received	64				
	BEP	68	0.773			
	Others (CP and EP)	59				

^a^Median value

^b^Iatrogenic or presurgical rupture

*CI* Confidence Interval, *BEP* Bleomycin Etoposide Cisplatin, *CP* Carboplatin Cisplatin, *EP* Etoposide Cisplatin

The median follow-up period of the patients was 87 months and ranged between 4 and 215 months. During this period, it was observed that 15 (13.4%) patients developed recurrence and 3 (2.7%) died becouse of the disease. Of the patients included in the study, the 5-year DFS was 85%, 10-year DFS was 83%. 5-year OS was 100% and the 10-year OS was 96%.

**Fig. 1:**
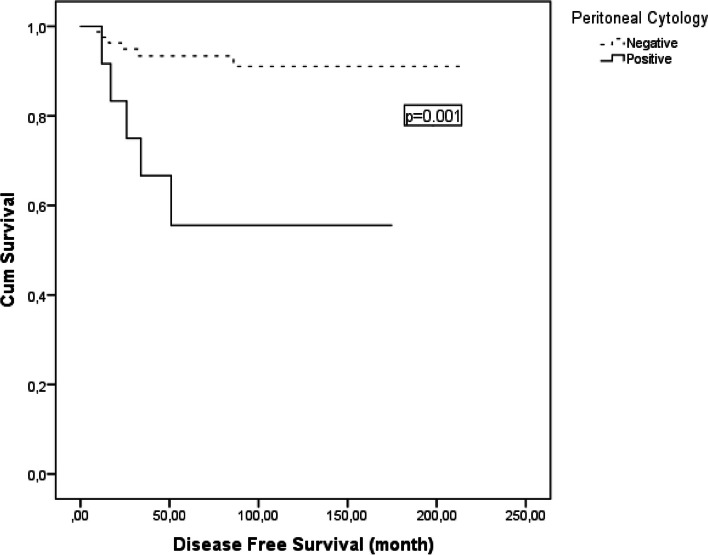
Cancer-specific survival of patients with granulosa cell tumors by peritoneal cytology

**Fig. 2:**
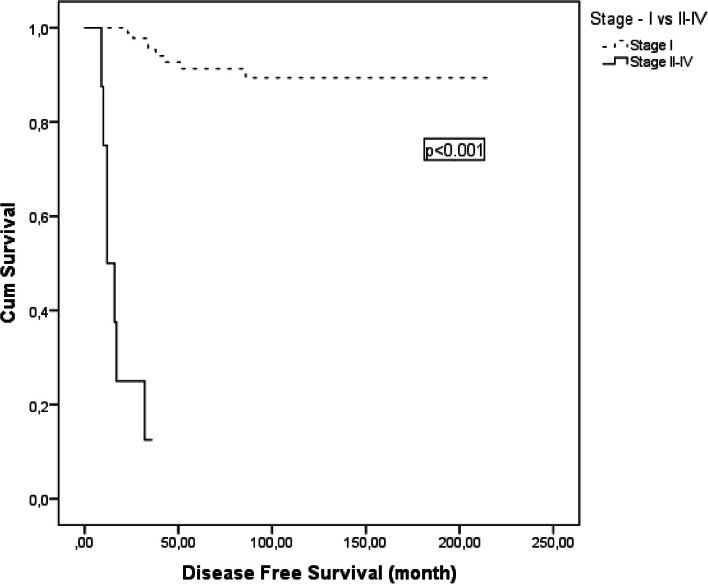
Cancer-specific survival of patients with granulosa cell tumors by FIGO stage

In the univariate analysis, positive peritoneal cytology, advanced stage and receiving of adjuvant treatment were associated with poor DFS. The 5-year DFS decreased from 93 to 56% in patients with positive peritoneal cytology (*p* = 0.001) (Fig. 1). The 5-year DFS which was 91% in stage 1, was 13% in stages 2–4 (*p* < 0.001) (Fig. 2). 5-year DFS significantly reduced in those receiving adjuvant therapy (respectively, 64% *vs*. 94%; *p* < 0.001) (Table 2.). However, this relationship was thought to be related to the stage of the disease, as treatment was mostly given to those experiencing stage IC-IV disease. In stage 1, 73.3% (*n* = 22/104) of patients received adjuvant treatment and all of them were in stage IC, whereas this rate was 100% (*n* = 8/8) in stages 2–4 (*p* < 0.001). Statistical analysis could not be performed for OS, as death due to disease occurred in only 3 patients.

Since adjuvant treatment was significantly correlated with stage, a model was created using peritoneal cytology and stage for multivariate analysis. Accordingly, stage 2–4 and positive peritoneal cytology were found to be independently poor prognostic factors for recurrence (respectively, odds ratio (OR) = 114.042, 95% confidence interval (CI) = 19.415–669.883, *p* < 0.001 and OR = 4.251, 95% CI = 1.125–16.072, *p* = 0.033) (Table 2.).

### Recurrence pattern

Of the 15 patients with recurrence, 7 (46.7%) had recurrence only in the pelvic region, 3 (20%) only in the upper abdominal region, 4 (26.6%) in the pelvic and upper abdominal region, and 1 (6.6%) in the upper abdominal and extra-abdominal region. Recurrences were observed as focal in 10 (66.6%) and as multifocal in 5 (33.3%) patients. The mean time for recurrence was 29 months and ranged between 9 and 86 months. Eight of the patients were in stage 1 and 7 were in stages 2–4.

Conservative surgery was applied in 1 patient (patient no: 1) at initial surgery. In addition adjuvant chemotherapy was given to 11 patients after initial surgery. Lymph node metastasis was present in 3 patients, omental metastasis in 4 patients and peritoneal cytology showed malignancy in 5 patients. Tumor-cyst rupture was present in 3 of the patients (Table 3).

**Table 3:** Clinico-pathological findings in patients with first recurrence after initial treatment (*n* = 15)

**Patient no**	**Age**	**FIGO Stage**	**Tumor size (mm)**	**Lymphadenectomy**	**Surgery type**	**Peritoneal cytology**	**Rupture of cyst**	**Adjuvant treatment**	**Time to recurrence (month)**	**Recurrence site**	**Secondary CRS**	**Salvage** **treatment**	**Clinical progress**
1	38	1C	65	Performed	Conservative	Malign	Iatrogenic rupture	BEP	51	Contralateral ovary,pelvic periton	Performed	BEP	CCR
2	78	1C	NR	Not performed	Definitive	NR	Unruptured	Not received	34	Sigmoid colon serosa	Performed	BEP	CCR
3	72	1B	55	Performed	Definitive	Benign	Unruptured	Not received	23	Pelvic	Performed	BEP	CCR
4	67	1C	160	Performed	Definitive	Benign	Unruptured	BEP	86	Sigmoid colon, ileum serosa	Performed	CP	CCR
5	52	1C	50	Performed	Definitive	Malign	Unruptured	Not received	26	Round ligamentum	Performed	BEP	CCR
6	37	1B	150	Performed	Definitive	Malign	Unruptured	Not received	34	Peritoneal	Performed	CP	CCR
7	43	2A	NR	Not performed	Definitive	NR	Unruptured	BEP	10	Pelvic ureter,omentum	Performed	BEP	CCR
8	61	2B	NR	Performed	Definitive	Benign	Unruptured	CP	32	Small bowel mesentery	Performed	CP	CCR
9	76	3C	260	Performed	Definitive	Benign	Unruptured	BEP	9	Lung, liver,small bowel mesentery	Not performed	BEP + EBRT	CCR
10	43	3B	200	Performed	Definitive	Benign	Unruptured	BEP	16	Omentum	Performed	EP	CCR
11	42	3C	220	Performed	Definitive	Malign	Unruptured	CP	17	Pelvic periton	Performed	Not received	CCR
12	57	4	160	Performed	Definitive	Benign	Unruptured	CP	12	Pelvic periton Glisson capsule	Performed	Not received	CCR
13	48	1C	130	Performed	Definitive	NR	Iatrogenic rupture	BEP	42	Peritoneal	Performed	BEP	CCR
14	47	1C		Not performed	Definitive	NR	NR	CP	38	Pelvic periton	Performed	CP	CCR
15	57	3B	120	Performed	Definitive	Malign	Presurgical rupture	CP	12	Omentum	Performed	BEP	CCR

*NR* Not reported, *BEP* Bleomycin Etoposide, Cisplatin, *CP* Carboplatin Cisplatin, *EP* Etoposide Cisplatin, *EBRT* External beam radiotherapy, *DOD* Died because of the disease, *AWOD* Alive without disease, *CCR* Complete clinical response

It has been determined that after the first recurrence, 14 (93.3%) of these patients underwent secondary cytoreductive surgery, followed by salvage chemotherapy. No residue tumor was observed in these patients after secondary cytoreduction. CCR was obtained with salvage treatment in all of these 14 patients. One patient, who developed extra-abdominal recurrence, received salvage chemotherapy and external radiotherapy (*patient no: 9*). After 18 months of follow-up, no recurrence has been observed yet in this patient. In finally, all patients with first recurrence CCR was achieved with salvage treatments after the first recurrence (Table 3).

It has been determined, that 6 of the patients developed a second recurrence and 5 patients with the secondary recurrence underwent tertiary cytoreductive surgery, followed by salvage chemotherapy (Table 4). No residue tumor was observed in these 5 patients after tertiary cytoreduction. One patient with extra-abdominal recurrence received salvage chemotherapy and external radiotherapy (*patient no 10*). CCR was achieved in 4 patients following salvage treatment after the second recurrence. Two patients with omental and extra-abdominal recurrence died due to progressive disease; the follow-up periods of these patients were 52 and 154 months after initial surgery, respectively (*patient no 2, and 10, respectively*).

**Table 4:** Clinicopathological findings in patients with multiple recurrences (*n* = 6)

Patient no	Second recurrence				Third recurrence				Follow-up time (month)	Last status
	**Recurrence** **site**	**Tertiary CRS**	**Salvage** **treatment**	**Clinical progress**	**Recurrence site**	**Quartinary CRS**	**Salvage** **treatment**	**Clinical progress**		
**2**	Pelvic, omentum	Performed	CP	PD					52	DOD
**4**	Pelvic	Performed	CP	CCR	Bladder wall, sigmoid colon	Performed	CP	CCR	106	AWOD
**6**	Peritoneal	Performed	BEP	CCR	Peritoneal	Performed	CP	CCR	87	AWOD
**7**	Pelvic,liver	Performed	CP	CCR	Liver,lung	Not performed	CP + EBRT	PD	83	DOD
**10**	Pelvic, lung	Not performed	CP + EBRT	PD					154	DOD
**13**	Spleen	Performed	CP	CCR					96	AWOD

*BEP* Bleomycin, Etoposide, Cisplatin, *EP* Etoposide, Cisplatin, *CP* Carboplatin, Cisplatin, *EBRT* External beam radiotherapy, *CRS* Cytoreductive Surgery, *CCR* Complete clinical response, *PD* Progressive disease, *DOD* Died because of the disease, *AWOD* Alive without disease

Furthermore, 3 of the 4 patients, with whom CCR was obtained after the second recurrence, developed a third recurrence and 2 of the patients underwent quaternary cytoreductive surgery, followed by salvage chemotherapy. No residue tumor was observed in these patients after quartenary cytoreduction. CCR was achieved in these two patients. The patient who were given salvage chemotherapy and external radiotherapy for extra-abdominal recurrence died due to progressive disease (*patient no 7*) (Table 4).

## Discussion

The clinical course of AGCT progresses slowly and the prognosis is good. Mangili et al. have reported 5-year DFS as 91.5%, 10-year DFS as 71.6% and 5-year OS as 97%, 10-year OS as 95% [11]. In our study with a median follow-up period of 87 months, 5-year DFS was 85%, 10-year DFS was 83% and 5-year OS was 100%, 10-year OS was 96%. Recurrence was observed in 13.4% of the patients. In multivariate analysis, advanced stage and positive peritoneal cytology were independent poor prognostic factors for DFS. Recurrence was 114 times higher in patients with stage 2–4 compared to stage I and 4.2 times higher in patients with positive peritoneal cytology compared to those with negative peritoneal cytology.

Stage is a well-defined prognostic factor associated with recurrence and survival in ACGT. Schumer et al. have reported 5-year OS to be 75–95% in stage I, 55–75% in stage II, 50% in stage III and 22% in stage IV [1]. Karalok et al. have reported that 5-year DFS was 96% in stage I, 70% in stage III and 50% in stage IV [6]. In our study, the 5-year DFS, which was 91% in stage 1, decreased to 13% in stages 2–4.

Guidelines from ESGO, SIOPE, and ESMO currently recommend the BEP regimen as the most commonly used regimen for advanced and recurrent AGCTs [12]. However, response rates for the conventional combination of bleomycin in recent studies are only between 22 and 35% [13]. The carboplatin/paclitaxel combination is emerging as a less toxic alternative to BEP.

Adjuvant chemotherapy is advocated especially in advanced stages and macroscopic residual disease [2, 10, 11]. Adjuvant chemotherapy may also be considered for extensive inoperable disease or recurrent disease. However, despite the high survival rate in AGCT, the role of adjuvant chemotherapy in the early stages is unclear. According to a recent meta-analysis, the administration of adjuvant chemotherapy did not improve the oncological and prognostic outcomes of *AGCT*, regardless of whether the patients had early or advanced/recurrent disease [13].

The development of new targeted drugs in conjunction with molecular studies in adjuvant treatment may increase the survival rates. Among the targeted drugs investigated for AGCT, antiangiogenic drugs have garnered attention. In a study by Tsoi et al., Bevacizumab (a monoclonal anti-VEGF-A antibody) treatment demonstrated reduced tumor growth and prolonged survival in AGCT [14]. But, in an randomized clinical trial of patients with relapsed SCST, adding bevacizumab to paclitaxel did not benefit [15]. New targeted approaches, such as tumor necrosis factor-related apoptosis-inducing ligand, FOXL2'nin (Forkhead box L2), nuclear factor kappa B (NF-kB), phosphatidyl inositol-3-kinase serine/threonine kinase pathway, and mammalian target of rapamycin (mTOR), may prove effective in treating AGCT [16].

Another adjuvant treatment option is radiotherapy. Evans et al.'s study found that radiotherapy had no significant effect on the relapse rate, with relapse occurring in 20% of patients receiving radiotherapy [17]. Similarly, Ohel et al. were unable to demonstrate any advantage in the use of radiotherapy for AGCT [18]. However, contrasting these findings, more recent and comprehensive studies have indicated that adjuvant radiotherapy (RT) can prolong survival in patients with advanced or recurrent AGCT disease. In the study by Hauspy et al., adjuvant RT resulted in a significantly longer disease-free survival (DFS) [19]. Moreover, in a recent comprehensive review by Barcellini et al., RT has shown promise and feasibility for unresectable AGCT and recurrent diseases [20]. The efficacy of radiotherapy in AGCT is not well defined due to limited data.

Positive peritoneal cytology is a controversial prognostic factor in AGCT. Especially in stage I (IC), it makes receiving adjuvant chemotherapy controversial. Lee et al. have found the positive peritoneal cytology rate of 11.8% in AGCT [21]. This rate was 10.7% in our study. In the studies presented by Lee et al. and Björkholm et al. peritoneal cytology positivity was found to be significant in terms of recurrence [9, 22]. In our study, the probability of recurrence was increased 4.2-fold in patients with positive peritoneal cytology and 5-year DFS decreased from 93 to 56%. On the contrary, in the studies of Park et al. and Ertas et al. no correlation has been demonstrated between peritoneal cytology positivity and recurrence [2, 23].

The incidence of lymph node metastasis at primary surgery in AGCT is low. Wang et al. have reported the incidence of lymph node metastasis as a 3.9% [24]. In our study, the rate of lymph node involvement was 3.2%. The addition of lymphadenectomy to the surgical procedure did not improve oncological outcomes. Similarly, Erkılınç et al. have also reported that lymphadenectomy did not lead to improvement in DFS and OS and, on the contrary, increased postoperative morbidity [25]. Abu-Rustum et al. have reported an isolated nodal recurrence rate of 5.9% and suggested that recurrences may be due to occult nodal metastases that were not detected at the time of the initial diagnosis [26]. Nevertheless, in the study presented by ourselves, no lymphatic recurrence was detected in any of the 15 patients with recurrence. In conclusion, removal of only suspicious lymph nodes rather than routine lymphadenectomy is the preferred surgical approach in AGCT.

Surgery is the primary treatment option for newly diagnosed or recurrent AGCT. However, the limits of primary surgery are not clear. Definitive surgery for early-stage primary tumors has been demonstrated to provide no survival or recurrence advantage compared with conservative surgery. The indications and the prognosis of the conservative approach are controversial [27]. In our study, conservative surgery did not worsen DFS rates when compared to definitive surgery. As for the advanced stage and recurrent tumors on the other hand, cytoreductive surgery is the most effective treatment method [8]. Sun et al. have stated that 85% of the patients with residual tumors developed recurrence [27]. In both primary and recurrent disease surgery, cytoreduction, having the goal of leaving no residual tumor, is important in terms of recurrence and DFS.

Due to the rarity of the disease, surgical experience data for AGCT recurrence is limited and there exists no consensus on how to choose the treatment. In the study by Lee et al. and Abu Rustum et al. most of the recurrences were intraperitoneal and 70% were pelvic [19, 21]. In our study, 93.3% of the primary recurrences were pelvic and intraabdominal and 6.6% were extra-abdominal. Recurrences in AGCT are usually focal and localized in one region [16]. In the study we presented, 66.6% of recurrences were focal and 33.3% were multifocal. This facilitates to avoid leaving residual tumor in salvage cytoreduction. Mangili et al. have reported that optimal debulking surgery is an effective treatment in case of recurrence [11]. However, recurring recurrences may develop during follow-up. In our study, surgeries have been performed on 14 out of 15 patients with recurrence, 4 out of 6 patients with second recurrence and 2 out of 3 patients with third recurrence without leaving residual tumor and complete clinical response has been obtained in all patients with the treatments offered. Whereas, 2 patients who could not undergo surgery due to extensive widespread disease died due to progressive disease in the second and third recurrence. In recurrent AGCTs, complete resection of the tumor determines survival outcomes [13]. If complete resection of the tumor can be achieved with salvage cytoreductions in recurrences, complete clinical response can be obtained in such patients.

Maximal cytoreductive surgery forms the cornerstone of treatment for primary and recurrent GCT. In cases of suboptimal surgical outcomes or unresectable metastatic disease, chemotherapy is commonly employed. However, there is limited data available on the use of adjuvant chemotherapy following complete cytoreductive surgery at recurrence [28, 29]. Surgery is recommended for patients with relapse according to ESGO guidelines. If the patient who has undergone complete debulking has not received chemotherapy afterward, follow-up or chemotherapy may be recommended. If one has received chemotherapy, the first option after surgery is follow-up, and the second is chemotherapy [30]. Yumru-Celiksoy et al. found that no benefit was derived from adjuvant systemic treatment, of any type, following complete cytoreductive surgery in patients with GCT-relapse [31]. A study by Memorial Sloan Kettering showed that chemotherapy did not improve the recurrence-free interval of patients with GCTs, even though also non-tumor-free operated patients were included [32]. In the multicenter retrospective MITO-9 study, further relapses were observed in 33% of patients who underwent surgery alone versus 37.5% of patients who underwent secondary cytoreductive surgery followed by chemotherapy. Mangili et al. noted that postoperative residual tumor was the only risk factor for decreased survival [11]. If surgery is performed without a tumor with repeated cytoreductive surgeries, the toxicity of unnecessary subsequent chemotherapy should be avoided [33].

İnhibin is secreted by granulosa cell tumors and is a useful tumor marker that falls after tumor removal and is also a marker for tumor recurrence. CA125 is not increased in GCTs, but sometimes it is useful in detecting relapse in those with values of Alfa-fetoprotein (AFP) / Beta-Human Chorionic Gonadotropin (β-hCG) within the normal range [25]. The production of estradiol by AGCT varies widely, and its value as a tumor marker is limited [34]. In the study by Haltia et al., HE4 and CA125 levels in AGCT patients were generally found to be below normal reference limits [35]. Rey et al. demonstrated that serum AMH can be considered as a marker for the diagnosis of ovarian AGCT [36]. Robertson et al. showed that inhibin levels were not elevated in all patients with AGCT and that serum inhibin was not specific for the diagnosis of AGCT [37]. Although the hormonal activity of ovarian AGCT suggests that the synthesized hormones may serve as tumor markers, the use of these tumor markers they have limited use in diagnosis and follow-up.

The retrospective nature of the study is the most important disadvantage. The relative high number of patients, a follow-up period of approximately 90 months, lymphadenectomy in 83.9% of the patients and the fact that the surgeries were performed without leaving residual tumors constitute the strengths of the study. In the present study, all the procedures have been performed by gynecooncologists and the pathology specimens have been evaluated by gynecopathologists as well.

## Conclusion

Advanced stage and peritoneal cytology are factors associated with survival and recurrence in AGCT. For appropriate eligible patients, offering of fertility-sparing approach at an early stage is a safe choice. Removal of suspicious lymph nodes should be preferred over systematic lymph node dissection. Most recurrences are curable with surgery and completion of surgery without leaving any residuals is the most important factor for survival. Since AGCT is rare and recurrence can occur at any stage; prospective, randomized, well-controlled and multicenter studies are required to clarify the prognostic factors.

## Data Availability

Data is provided within the manuscript or supplementary information files.
